# Plant Wearable Sensors Based on FBG Technology for Growth and Microclimate Monitoring

**DOI:** 10.3390/s21196327

**Published:** 2021-09-22

**Authors:** Daniela Lo Presti, Sara Cimini, Carlo Massaroni, Rosaria D’Amato, Michele Arturo Caponero, Laura De Gara, Emiliano Schena

**Affiliations:** 1 Unit of Measurement and Biomedical Instrumentations, Departmental Faculty of Engineering, Università Campus Bio-Medico di Roma, Via Alvaro del Portillo, 21, 00128 Rome, Italy; c.massaroni@unicampus.it (C.M.); e.schena@unicampus.it (E.S.); 2 Unit of Food Science and Nutrition, Department of Science and Technology for Humans and the Environment, Università Campus Bio-Medico di Roma, Via Alvaro del Portillo, 21, 00128 Rome, Italy; s.cimini@unicampus.it (S.C.); l.degara@unicampus.it (L.D.G.); 3 Photonics Micro and Nanostructures Laboratory, Fusion and Technologies for Nuclear Safety and Security Department, FSN-TECFIS-MNF, ENEA C.R. Frascati, Via E. Fermi, 45, 00044 Frascati, Italy; rosaria.damato@enea.it (R.D.); michele.caponero@enea.it (M.A.C.)

**Keywords:** plant wearable sensors, fiber Bragg grating sensors, fiber optic sensors, plant growth monitoring, microclimate monitoring, strain sensing, temperature sensing, humidity sensing

## Abstract

Plants are primary resources for oxygen and foods whose production is fundamental for our life. However, diseases and pests may interfere with plant growth and cause a significant reduction of both the quality and quantity of agriculture products. Increasing agricultural productivity is crucial for poverty reduction and food security improvements. For this reason, the 2030 Agenda for Sustainable Development gives a central role to agriculture by promoting a strong technological innovation for advancing sustainable practices at the plant level. To accomplish this aim, recently, wearable sensors and flexible electronics have been extended from humans to plants for measuring elongation, microclimate, and stressing factors that may affect the plant’s healthy growth. Unexpectedly, fiber Bragg gratings (FBGs), which are very popular in health monitoring applications ranging from civil infrastructures to the human body, are still overlooked for the agriculture sector. In this work, for the first time, plant wearables based on FBG technology are proposed for the continuous and simultaneous monitoring of plant growth and environmental parameters (i.e., temperature and humidity) in real settings. The promising results demonstrated the feasibility of FBG-based sensors to work in real situations by holding the promise to advance continuous and accurate plant health growth monitoring techniques.

## 1. Introduction

Plants are crucial to the existence of all living organisms on Earth [1]. They provide for 98% of the oxygen we breathe and, being the basis of the trophic network, for 80% of the food we eat. Plants are also primary sources for drugs, textile fibers, and other essentials for human life, and they are pivotal in the interactions among the environment, humans, and animals [2]. Unfortunately, insect abundance and abiotic factors, including salinity, radiation, and extremes in temperature (T) and relative humidity (RH) levels may have strong impacts on the survival rate of the plants [3,4,5,6,7].

The Food and Agriculture Organization (FAO) estimated that 40% of crops productivity is lost annually due to plant pests, diseases, and environmental stresses related to climate changes which are seriously damaging agriculture, people’s nutrition, and food security [8,9].

To reduce crop losses and poverty, the 2030 Agenda with the Sustainable Development Goals recognizes the central role of agriculture in increasing competitiveness and productivity at the plant level in a sustainable way [10,11].

To accomplish these goals, the agriculture sector is experiencing a revolution called Smart Farming, where novel sensors are combined with image processing, big data, and cloud computing to measure the plants’ growth and environmental parameters such as T, RH, solar radiation, and mineral nutrition [12,13,14].

The state-of-the-art technologies used for quantitatively measuring plant physiology and environmental factors are based on contactless methods such as spectroscopy, airborne/satellite imagery, and machine vision systems [13,15,16,17,18,19,20]. However, the lack of high spatial and temporal resolution of these systems and their discrete measurements do not allow the continuous and accurate monitoring of plants’ health as well as the effects of biological/environmental factors on their growth [21,22].

In contrast to conventional methods, recently, novel techniques like wearables and skin-mountable devices developed for human beings have been extended to plants [14,23]. In particular, thanks to the advance of flexible electronics, small, stretchable, and miniaturized sensors have been developed to be directly attached to the plants or printed on their leaves for monitoring the plant’s health and microclimate changes [14,24,25,26,27,28,29].

An attractive solution that may offer unique strengths for developing plant wearables is the fiber Bragg grating (FBG) sensing technology. FBG sensors are popular in several fields, from human health monitoring [30,31,32,33,34,35] to aerospace industry [36,37,38], civil engineering [39,40,41], and biosensing [42,43], thanks to many advantages like the FBG high sensitivity, small size, and lightweight. Although these features make the FBG technology particularly suitable for plant sensing, they are still overlooked for applications in the agriculture sector. Moreover, the FBG intrinsic sensitivity to strain (*ε*), and T variations (Δ*T*), the potential grating functionalization with a moisture-activated polymer to detect RH changes (ΔRH) in the surrounding air, as well as their multiplexing capability may offer unique features to miniaturized sensing solutions for the simultaneous monitoring of both plant growth and environmental conditions [33,44,45,46].

Here, we propose an innovative system consisting of three plant wearable sensors based on FBG technology for monitoring the growth and the microclimate (i.e., T and RH) of tomato plants chosen as a model of a plant of agronomic interest. The assessment of the proposed sensors was carried out in real settings: inside a growth chamber under controlled T and RH levels, and outdoors in a field. To the best of our knowledge, this is the first attempt to develop FBG-based plant wearables for applications in Smart Farming.

The remainder of the present study is structured as follows: Section 2 focuses on the plant wearables’ design, fabrication, and their working principles. Section 3 describes the metrological properties of the developed sensors. Section 4 proposes the feasibility assessment of the plant wearables in two scenarios of application: inside a growth chamber and in-field (outdoors). Finally, discussions and conclusions are included in Section 5 and Section 6, respectively.

## 2. The FBG-Based Plant Wearables: Design, Fabrication, and Working Principles

The proposed system consists of three sensors:

- a dumbbell-shaped flexible sensor to be directly attached to the plant stem for monitoring its growth via *ε* sensing;
- an environmental sensor consisting of a chitosan (CH)-coated FBG sensor for RH monitoring;
- an environmental sensor consisting of a bare FBG for T monitoring.

The three sensors were multiplexed together in an array configuration to reduce the overall system encumbrance, and an FC/APC connector was joined at the end of the optical fiber for enabling the FBG’s interrogation.

**Figure 1:**
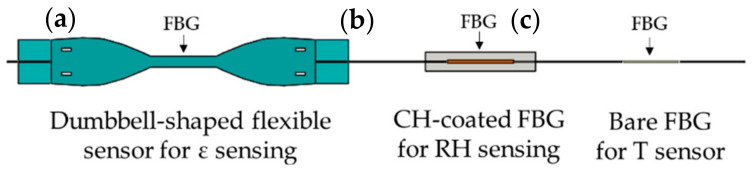
Schematic of the plant wearables in an array configuration: (**a**) the dumbbell-shaped flexible sensor for *ε* sensing, (**b**) the CH-coated FBG for RH monitoring, and (**c**) the bare FBG for T measurements.

Figure 1 shows a schematic representation of the proposed plant wearables.

An FBG is a microstructure inscribed into the core of an optical fiber. It works as a notch filter that back-reflects a narrow portion of light centered around the so-called Bragg wavelength (*λ_B_*) when enlightened by a broad spectrum of light. Its working principle is based on the Bragg condition [47]:

(1) $$\lambda_{B}=2\eta_{eff}$$

where *η_eff_* is the effective refractive index of the fiber core and Λ, the grating period. Generally, both these parameters change in accordance with Δ*T* and *ε* applied along the longitudinal axis of the optical fiber following:

(2) $$\frac{\Delta \lambda_{B}}{\lambda_{B}}=\left(1-p_{e}\right)\cdot \varepsilon +\left(\left(1-p_{e}\right)\cdot \alpha_{\Lambda }+\alpha_{n}\right)\cdot \Delta T)$$

with *p_e_* the effective strain optic coefficient, *α_Λ_* the fiber thermal expansion coefficient, and *α_n_* the fiber thermo-optic coefficient.

In the following sections, we describe the design of each wearable sensor with a focus on the main fabrication steps and their principles of work.

### 2.1. The Plant Wearable Sensor for Growth Monitoring

The plant wearable sensor for growth monitoring consists of a commercial FBG (grating length of 10 mm, *λ_B_* of 1533 nm with acrylate recoating, AtGratings Technology, Shenzhen, China) encapsulated into a flexible matrix made of Dragon Skin™ 20 (Smooth-On, Macungie, PA, USA). The use of a high-stretchable material improves the FBG robustness and adherence to the plant, making its anchorage to the stem easier.

The proposed system has a dumbbell shape to improve the FBG sensitivity to *ε* (S*_ε_*) with overall dimensions of 48 mm × 8 mm × 1 mm and a narrow part of 12 mm × 2 mm × 1 mm.

To obtain this shape, a mold was firstly designed in Solidworks 2019 (Dassault Systemes Solidworks Corp, Waltham, MA, USA) and then 3D printed using Ultimaker 2+ (Ultimaker, Utrecht, The Netherlands).

The main fabrication steps necessary to develop the dumbbell-shaped flexible sensor are listed below:

1. the FBG sensor is placed inside the mold;
2. the silicone mixture is prepared: Dragon Skin™ 20 silicone part A and part B are equal weight mixed and then vacuum degassed to remove the trapped air bubbles;
3. the mixture is poured in the mold to cover the FBG sensor and the curing process starts. After 4 h at room temperature, the plant wearable sensor is ready for use.

**Figure 2:**
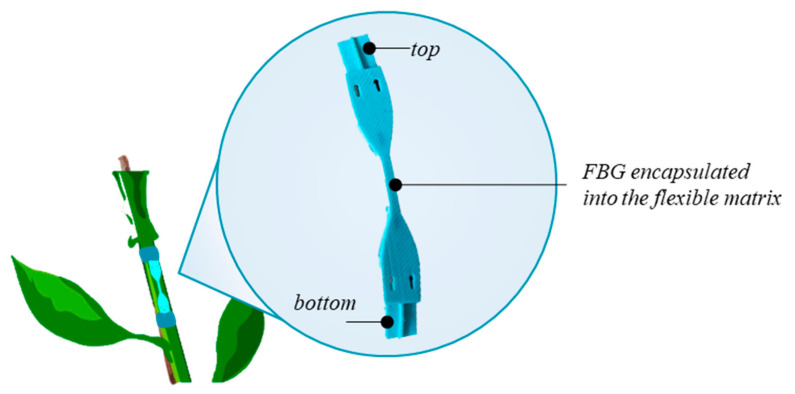
Schematic representation of the developed dumbbell-shaped flexible sensor anchored around the plant stem.

The final structure allows the sensor to be easily mounted on the plant by wrapping the top and the bottom parts around the stem (see Figure 2).

Once compliant to the stem, the sensor experiences Δ*λ_B_* increments when strained and decrements when compressed (see Equation (2)). During its elongation, the plant pulls the flexible matrix and, in turn, strains the encapsulated grating by inducing a shift of *λ_B_* (i.e., Δ*λ_B_*).

### 2.2. The Environmental Plant Wearable Sensors for Microclimate Monitoring

In this study, we developed two environmental plant wearable sensors to monitor RH and T since their variations can influence the plant microclimate and indirectly affect the crop quality and plants’ growth.

The RH sensor was developed by functionalizing a commercial FBG sensor (length of 10 mm, *λ_B_* of 1541 nm with acrylate recoating, AtGratings Technology, Shenzhen, China) with a CH coating. CH is a polysaccharide produced from the deacetylation of chitin, a biopolymer from the exoskeletons of crustaceans and insects [48].

The CH coating was deposited on the FBG sensor as a solution prepared by dissolving low molecular weight CH with a concentration of 5% wt. in 2% *v*/*v* aqueous solution of acetic acid following the procedure in [49]. All chemicals were reagent grade from Sigma-Aldrich^®^, St. Louis, MO, USA). The CH solution was kept overnight to remove the air bubbles trapped during the gel preparation. The day after, 0.15 g of the CH gel was used to coat the FBG sensor, placed on a filter paper, and dried at room temperature for 12 h.

The CH gel swelling behavior in response to changes in the water content in the air surrounding the functionalized FBG was exploited to make the sensor sensitive to RH (S_RH_).

When the content of water vapor increases, the CH coating swells, strains the FBG and in turn, causes an increment of *λ_B_*. Otherwise, a reduction of the water vapor content induces shrinkage of the CH coating and, in turn, a decrement of *λ_B_*. The mathematical description of such behavior is the following [50]:

(3) $$\frac{\Delta \lambda_{B}}{\lambda_{B}}=\left(1-p_{e}\right)\cdot \varepsilon_{RH}+\left(\left(1-p_{e}\right)\cdot \alpha_{\Lambda }+\alpha_{n}\right)\cdot \Delta T)$$

with $\varepsilon_{RH}$ the elongation along the longitudinal axis of the fiber due to CH volumetric changes induced by RH variations.

Regarding the T sensor, we exploited the FBG intrinsic sensitivity to T (S_T_), as shown in Equations (2) and (3), by multiplexing a bare FBG to the other two wearables (i.e., the flexible sensor and the CH-coated FBG sensor).

**Figure 3:**
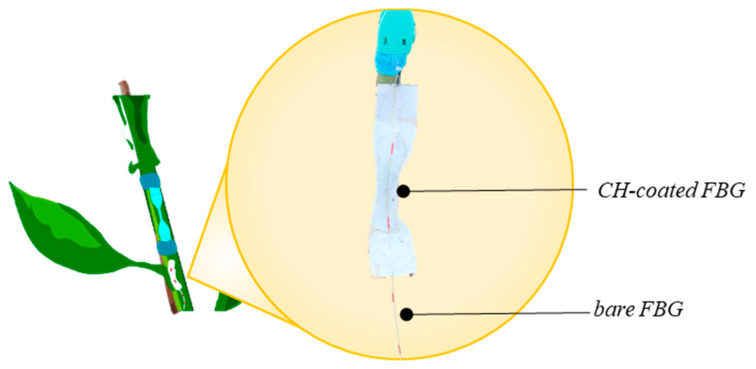
Schematic representation of the environmental plant wearable sensor: the CH-coated FBG sensor in the filtered paper for RH sensing and the bare FBG sensor for T sensing.

A schematic representation of the environmental plant wearables is shown in Figure 3.

## 3. Metrological Characterization of the Plant Wearable Sensors

The proposed wearable sensors were developed to monitor simultaneously and continuously the plant growth and the localized microclimate (T and RH levels) around the plants.

Before their use in the scenario of interest, a metrological characterization was performed to investigate the response of the dumbbell-shaped flexible sensor to *ε* in terms of S*_ε_* and the response of the CH-coated FBG sensor to RH in terms of S_RH_.

Regarding the S_T_ of the bare FBG, we used the nominal value provided by the manufacturer (i.e., ~0.01 nm·°C^−1^).

### 3.1. Strain Sensitivity of the Dumbbell-Shaped Flexible Sensor

To estimate S*_ε_*, a static assessment of the dumbbell-shaped plant wearable sensor was carried out by using a tensile testing machine (Instron, mod. 3365). The sensor was placed between the lower and upper grips of the machine with an initial length (l_0_) of 12 mm.

The machine was set to apply a controlled *ε* from ~0% up to 2% of l_0_ at quasi-static conditions (i.e., at a low displacement rate of 1 mm·min^−1^) and room temperature.

**Figure 4:**
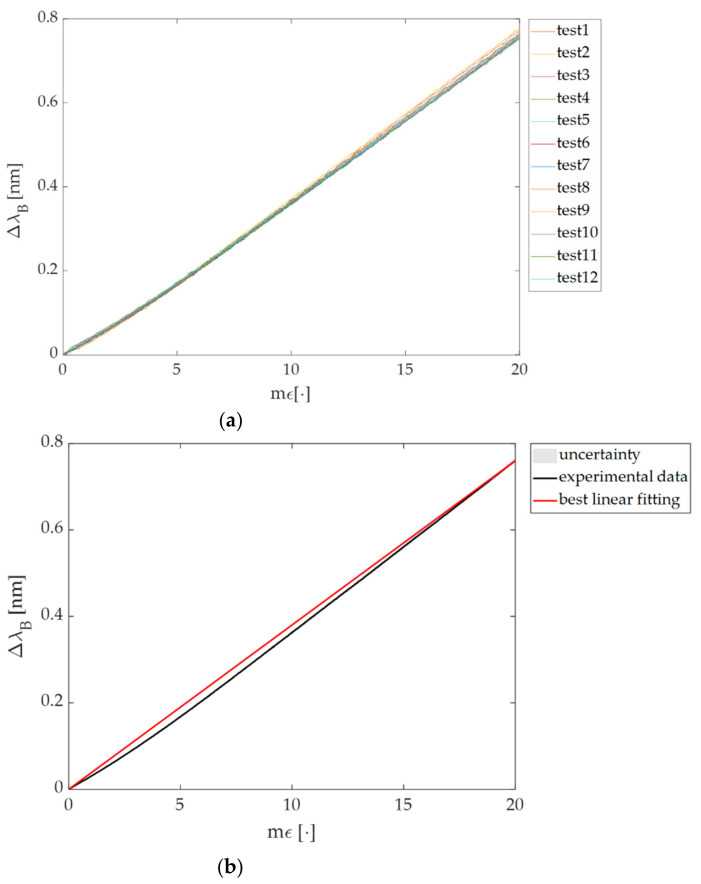
(**a**) The response of the flexible sensor across the twelve tests and (**b**) the calibration curve Δ*λ_B_* vs. m*ε* obtained by the experimental data (black line) with the best fitting line in red.

Data from the tensile machine were recorded at the sampling frequency of 100 Hz while the Δ*λ_B_* of the FBG-based plant wearable sensor were collected by the optical spectrum interrogator (si255 based on HYPERION platform; Micron Optics Inc., Atlanta, GA, USA) at the same sampling frequency. The test was repeated twelve times to investigate the repeatability of the system response (i.e., Δ*λ_B_*) to the applied *ε*. Figure 4a shows the Δ*λ_B_* vs. *ε* trends of all the mechanical tests. In both Figure 4a,b, the applied *ε* is expressed in terms of m*ε* because usually the S*_ε_* measurement unit of an FBG sensor is nm·m*ε*^−1^.

Experimental data were processed through a custom algorithm to extract the calibration curve (Δ*λ_B_* vs. *ε*) and the associated uncertainty in the MATLAB environment.

The calibration curve was considered as the best linear fitting of the mean Δ*λ_B_* response to *ε* over the twelve tests. The expanded uncertainty was estimated by multiplying the standard uncertainty by the coverage factor (k = 2.20) obtained from a Student’s t-distribution with eleven degrees of freedom and a 95% confidence interval as reported in Figure 4b.

Finally, considering the linearity of response, S*_ε_* was considered equal to the slope of the best fitting line (i.e., 0.04 nm·m*ε*^−1^) as shown in Figure 4b. The goodness of the linear fit is testified by R^2^ > 0.99.

### 3.2. Relative Humidity Sensitivity

To retrieve S_RH_, the CH-based FBG sensor was exposed to RH slow changes (from 10% to 90%) inside a custom climatic chamber to extensively cover the operating RH range of the proposed sensing element.

A capacitive RH sensor (HIH-4000-002, accuracy of ±3.5%RH, Honeywell International Inc., Charlotte, NC, USA) was used as a reference instrument.

Both Δ*λ_B_* and the reference RH values were collected at 100 Hz. The FBG outputs were recorded by using an FBG interrogator (si255 based on HYPERION platform, Micron Optics) while the output of the reference instrument was collected by using a data acquisition board (NI DAQ USB-6009, Austin, TX, USA) and a LabView interface to track in real-time the RH level reached inside the chamber.

To cover the range of RH from 10% to 90%, an airflow delivered by a mass flow controller (EL-Flow, Bronkhorst High-Tech, Ruurlo, The Netherlands) at 2 L·min^−1^ and humidified by a heated humidifier (MR850, Fisher & Paykel Healthcare, Auckland, New Zealand) was forced inside the chamber.

The collected data were analyzed in a MATLAB environment to retrieve the calibration curve (Δ*λ_B_* vs. %RH) and then, the value of S_RH_.

**Figure 5:**
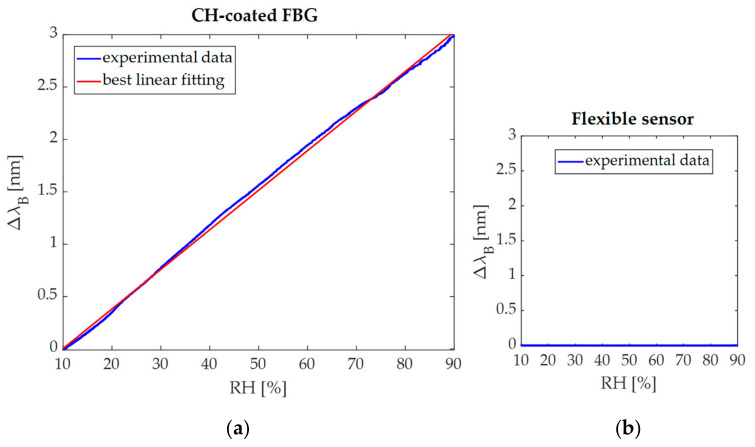
(**a**) Calibration curve Δ*λ_B_* vs. RH of the CH-coated FBG and (**b**) the negligible influence of RH on the output of the dumbbell-shaped flexible sensor.

Figure 5a shows the linear response of the CH-coated FBG sensor to RH (R^2^ > 0.99) with a S_RH_ of 0.04 nm·%RH^−1^.

The output of the dumbbell-shaped flexible sensor was also collected during the experimental test in order to assess the negligible effect of RH on its output changes. This result is shown in Figure 5b.

## 4. Feasibility Assessment of the Plant Wearable Sensors

This section proposes the experimental setup and protocol followed for the acquisitions inside the growth chamber and outdoors in Section 4.1; data analysis and results of the performed acquisitions in Section 4.2.

### 4.1. Experimental Setup and Protocol

The preliminary assessment of the proposed sensors was carried out in two real scenarios: in a plant’s growth chamber to assess the sensors’ performance in a controlled environment and outdoors to investigate the in-field working capability of the plant wearable sensors of monitoring both elongation and environmental conditions.

In both cases, a tomato plant was instrumented with the proposed sensing elements and monitored for ~12 h in the growth chamber and ~22 h during the in-field acquisitions.

#### 4.1.1. The Growth Chamber Acquisition

The feasibility of plant wearable sensors to monitor the elongation of the tomato plant and its microclimate was firstly assessed in a growth chamber (Binder KBWF 720, accuracy 0.3 °C and ± 1.5%, Tuttlingen, Germany,) at a fixed T of 25 °C, RH of 60%, and light of 120–150 μmol ·m^−2^·s^−1^ (that corresponds to ~6500 lux–~8100 lux considering a sunlight source). The acquisition lasted ~12 h.

A tomato plant was equipped with the plant wearables, and the flexible sensor was anchored around its stem.

**Figure 6:**
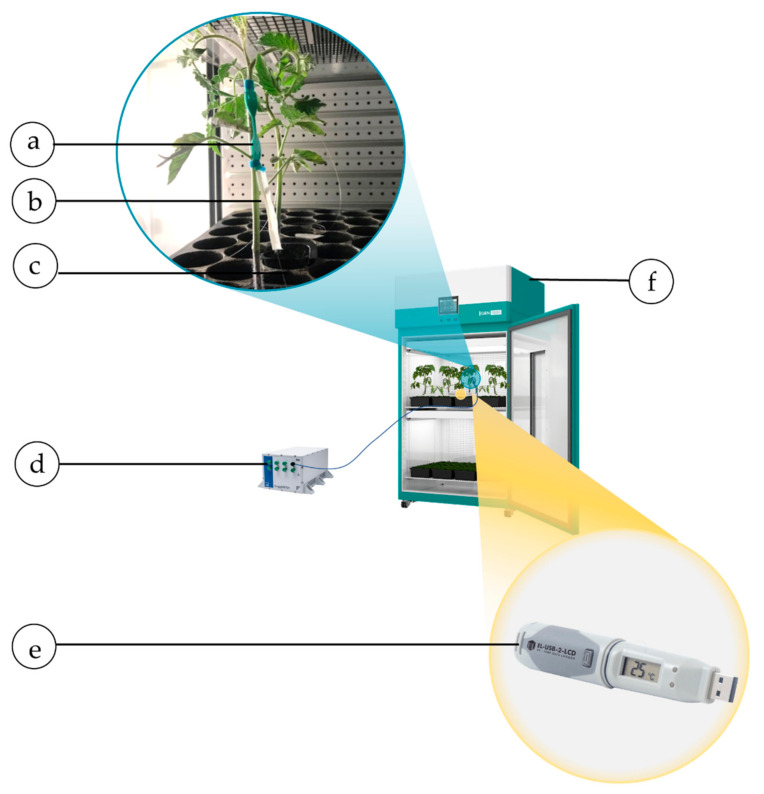
Experimental setup for test inside the growth chamber: the plant wearable sensor mounted on the stem (**a**), the CH-coated FBG sensor (**b**), and the bare FBG sensor (**c**), the FBG interrogator (**d**), and the reference probe for measuring T and RH levels (**e**), and the growth chamber (**f**).

Data from the wearables were collected using the FBG interrogator (FS22, HBM FiberSensing, Bedford, UK) at 1 Hz. Simultaneously, a reference instrument (EL-USB-RT, Temperature & Humidity probe, accuracy ± 1.5 °C, and ±4.5%RH, Lascar Electronics, Eire, PA, USA) was used to record T and RH reference values at a sampling frequency of ~0.0167 Hz (i.e., one sample per minute). The experimental setup is shown in Figure 6.

#### 4.1.2. The Outdoor In-Field Acquisition

The outdoor assessment was carried out to preliminarily investigate the performance of the proposed wearable sensors in a real setting under uncontrolled environmental conditions.

A tomato plant was placed in a field localized in Rome (Italy) and then equipped with the developed sensors.

**Figure 7:**
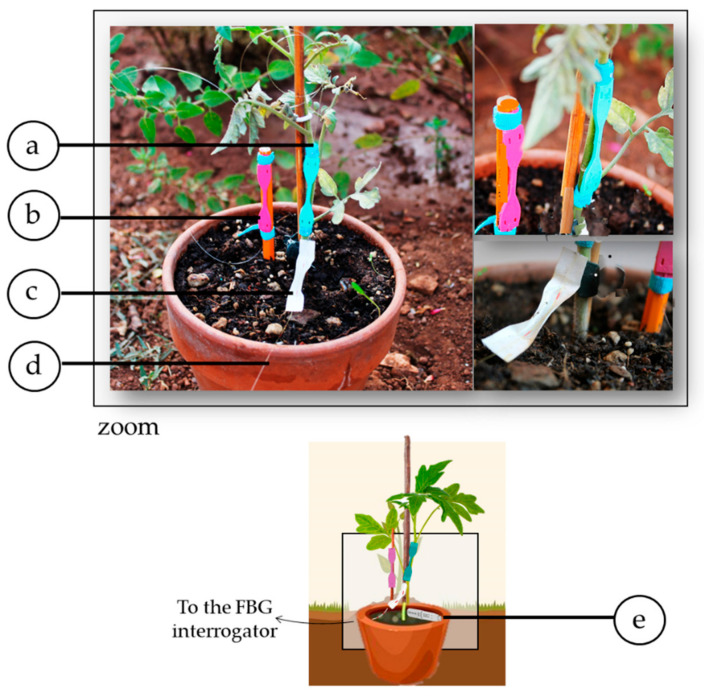
In-field experimental setup: the flexible sensor around the plant stem (**a**) and the one around the support stick (**b**); the CH-coated FBG (**c**) and the bare FBG (**d**) with the reference instrument (**e**). A zoom on the plant wearables and pictures in a real setting are also reported.

Considering the challenging scenario and the potential influence of other unpredictable factors (e.g., wind blowing, animal–plants interactions, rain), we developed a wearable sensor nominally identical to the one attached to the stem but mounted on a wooden stick placed in the proximity of the instrumented plant (see Figure 7).

The Δ*λ_B_* values of the wearable sensor placed on the stick recorded during the in-field acquisition were useful to compensate the output changes of the flexible plant wearable sensor due to external factors such as Δ*T* and ΔRH in order to emphasize the Δ*λ_B_* contributions induced by the plant elongation.

The same instruments already described for the growth chamber acquisition were used to collect data from the plant wearable sensors and the FBG-based flexible sensor placed around the wooden support outdoors and the reference T and RH values. The FBG interrogator (FS22, HBM FiberSensing) was placed at ~ 20 m from the installed setup and connected to a laptop for checking data in real-time. A patch cord was used for connecting the sensors to the FBG interrogator while the reference instrument (EL-USB-RT, Temperature & Humidity probe, Lascar Electronics, Eire, PA, USA) was placed close to the monitored plant.

### 4.2. Data Analysis and Results

Data collected both inside the growth chamber and during the in-field acquisition were analyzed in the MATLAB environment by following two main steps: (i) data synchronization and resampling; (ii) T compensation of data collected by the dumbbell-shaped flexible sensor and the CH-coated FBG sensor by removing the Δ*λ_B_* values experienced by the bare FBG during the whole test duration, intrinsically sensitive to T.

The obtained results are described starting from the ones related to the growth chamber acquisition.

#### 4.2.1. The Growth Chamber Acquisition

**Figure 8:**
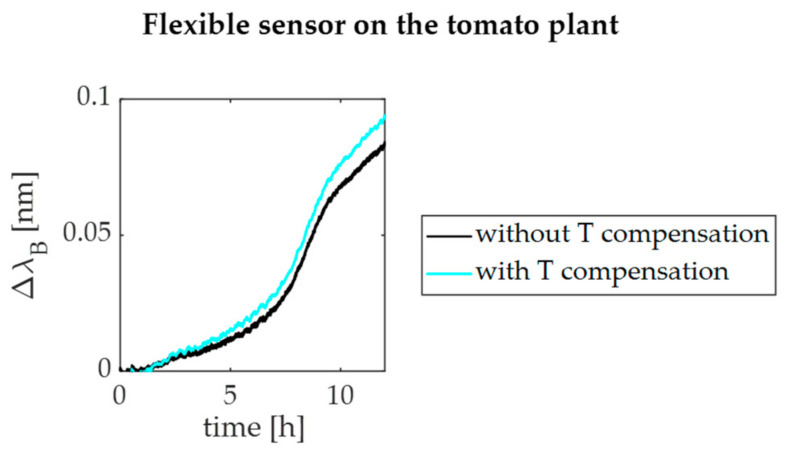
The T non-compensated (black line) and T-compensated (cyan line) output changes of the flexible sensor mounted on the stem under controlled T and RH levels.

Focusing on *ε* sensing, the response of the plant wearable flexible sensor mounted on the stem is shown in Figure 8.

Results showed that the dumbbell-shaped flexible sensor experienced an elongation over the 12 h lasting acquisition. Indeed, under controlled environmental conditions inside the growth chamber, the T-compensated sensor output reached Δ*λ_B_* of ~0.1 nm. This value corresponds to an elongation of ~120 μm, quantified by exploiting the S*_ε_* value obtained by the Δ*λ_B_* vs. *ε* calibration curve in Figure 4b.

**Figure 9:**
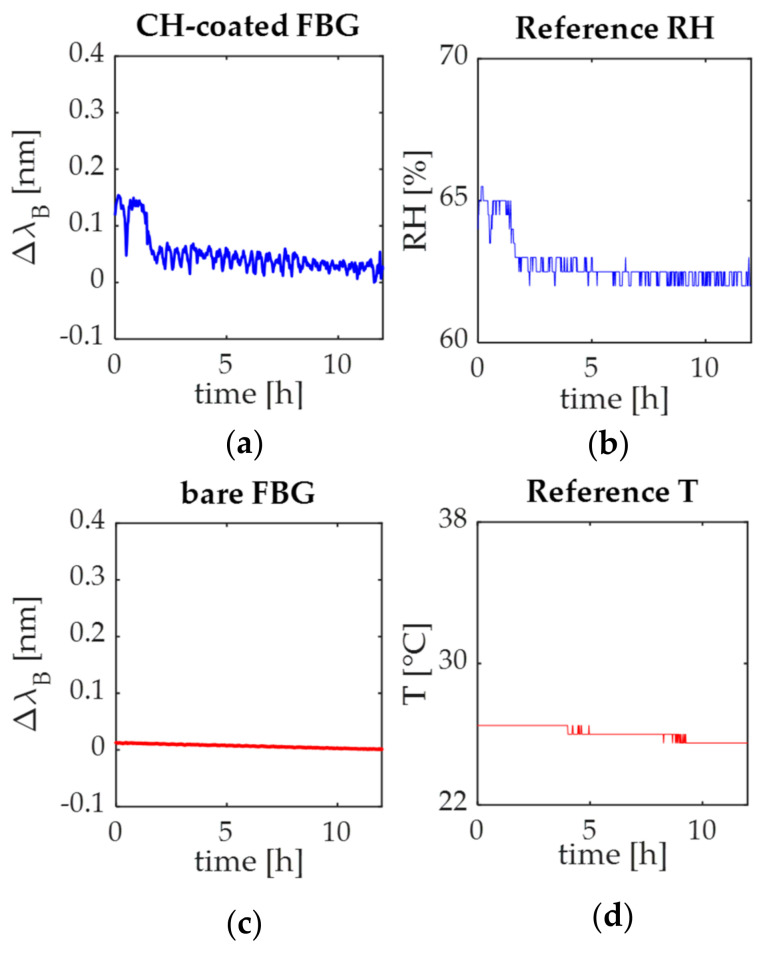
(**a**) the CH-coated FBG output changes; (**b**) the reference RH signal; (**c**) the bare FBG signal; (**d**) the reference T signal.

The responses of the environmental plant wearables and the reference ones are shown in Figure 9a–d.

In both cases, the plant wearables for RH and T monitoring showed the same trend of the reference signals.

Focusing on RH sensing, the slight variations in the reference RH signal (Figure 9b) are related to the measurement accuracy declared by the chamber manufacturer and the reference T and RH probe. These changes led to a maximum output change of ~0.15 nm when RH reached ~65% (Figure 9a,b).

Regarding the T monitoring, results confirmed that the T value was kept constant inside the chamber (i.e., ~25 °C as shown in Figure 9c) over the whole acquisition, leading to negligible changes of the bare FBG during the whole acquisition (Figure 9d).

#### 4.2.2. The Outdoor In-Field Acquisition

**Figure 10:**
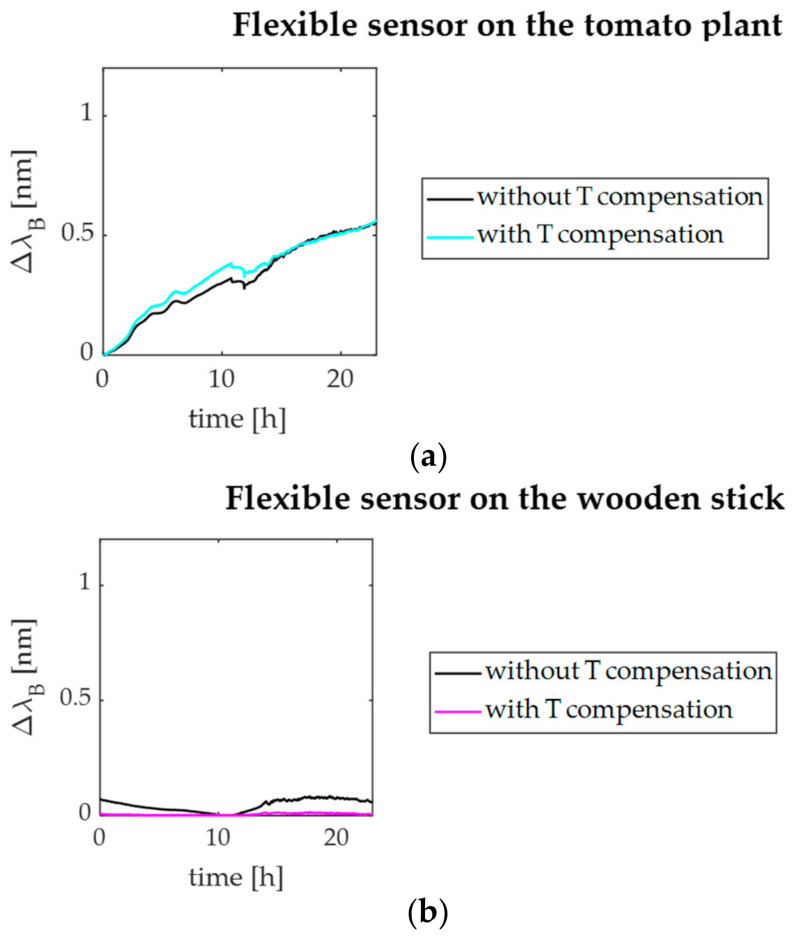
Signals recorded during the in-field experiment by the flexible sensors: (**a**) the T non-compensated (black line) and T-compensated (cyan line and magenta lines) output of the dumbbell-shaped flexible sensor mounted on the plant, and (**b**) the ones of the flexible sensor mounted on the stick.

Results of the outdoor in-field acquisition are reported in Figure 10.

In particular, the output changes of the flexible sensor mounted on the stem and those of the sensor around the support stick are plotted in Figure 10a,b, respectively, in terms of both T non-compensated (black lines) and T-compensated responses.

As shown in Figure 10a, even after the T compensation, considerable Δ*λ_B_* increments were experienced by the plant wearable sensor around the stem over the ~22 h lasting acquisition. This value corresponds to an elongation of ~720 μm.

**Figure 11:**
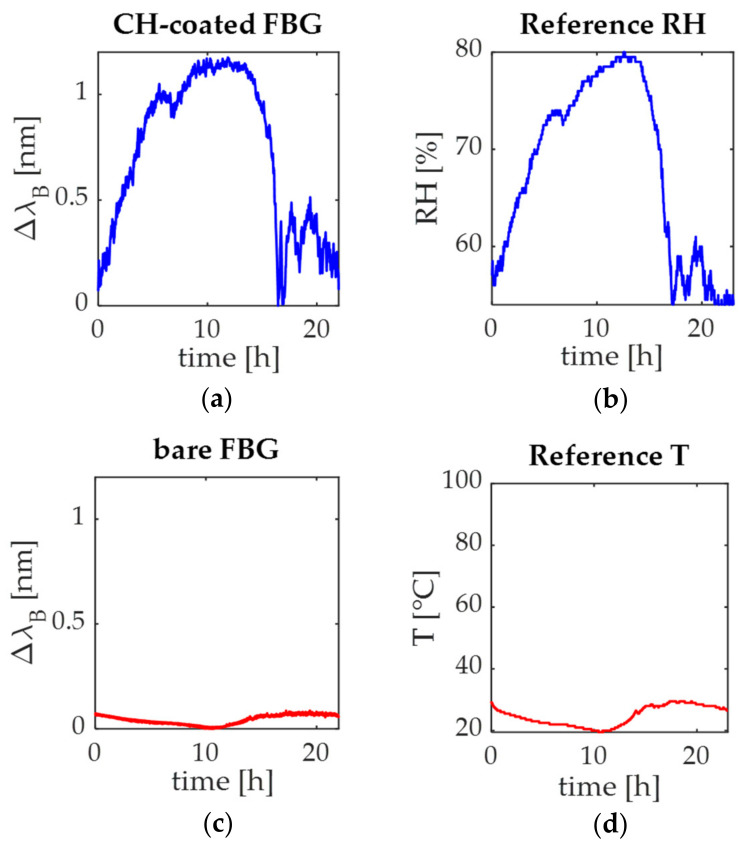
Signals recorded during in-field experiments by the environmental sensors: (**a**) the output changes of the CH-coated FBG sensor with (**b**) the reference RH signal; (**c**) the bare FBG signal; (**d**) the reference T signal.

Differently, the response of the sensor placed on the stick had a trend (black line in Figure 10b) similar to the one of the bare FBG (Figure 11b). Therefore, the T-compensated Δ*λ_B_* values showed no considerable variations over time (magenta line in Figure 10b).

These findings confirmed the capability of the developed flexible plant wearable sensor mounted on the stem to monitor the plant growth even during in-field long acquisitions when any control of environmental parameters is performed.

Focusing on the plant wearable environmental sensors, their output changes over time are reported in Figure 11a,c together with the T and RH signals collected by the reference instrument (Figure 11b,d).

Results showed that the trend of both the CH-coated and the bare FBG sensors followed those of the reference RH and T signals, respectively: at night, when RH decreases, T increases (RH moves from 56% to ~80% and the CH-coated Δ*λ_B_* from ~0 nm to ~1 nm while T changes from 29 °C to ~20 °C and Δ*λ_B_* of the bare FBG sensor from ~0.09 nm to ~0 nm) and vice versa during the day (RH moves from 80% to 52% and the CH-coated Δ*λ_B_* from 1 nm to ~0 nm while T moves from 20 °C to 27 °C and Δ*λ_B_* of the bare FBG sensor from ~0 nm to ~0.07 nm).

## 5. Discussion

Here, we proposed plant wearable sensors based on FBG technology for the simultaneous monitoring of plant growth and its microclimate (i.e., T and RH). This is the first study that investigated the feasibility of FBG-based plant wearables to continuously monitor stem longitudinal elongation simultaneously to localized environmental parameters.

Following this aim, we took advantage of some distinctive features of FBG-sensing technology to develop small, light, highly performant, and stretchable sensors suitable for harmlessly cohabitating with the plants and monitoring their health status. A flexible sensor was comfortably wrapped around the stem and used for monitoring the longitudinal plant growth via *ε* sensing, and the two environmental sensors, a CH-coated FBG, and a bare FBG, were placed at the plant level to monitor RH and T, respectively. The sensors’ sensitivity values were retrieved (S*_ε_* of 0.04 nm·m*ε*^−1^, S_RH_ of 0.04 nm·%RH^−1^, and S_T_ of 0.01 nm·°C^−1^) before their preliminary assessment in real settings (inside a growth chamber and outdoors in a field) with promising results (as already described in Section 5).

In the literature, the combined measurement of plant growth and environmental factors has been recently proposed for increasing the survival rate of plants and augmenting the quality of agriculture outputs. All the wearable systems involved in these studies consist of solutions based on flexible and printable electronics [16,23,24,25,27,51].

A few studies have proposed solutions for monitoring plant growth focusing on fruit expansion [24,51]. However, they did not provide concrete information about the health parameters and growth of the plant.

Otherwise, recent studies proposed multisensory platforms, multifunctional wearable and printable sensors for monitoring stem or leaf growth, and the effect of several stressing factors (e.g., T and RH levels in the surrounding air, incident sunlight, and water resources [16,23,25,27]) on the growth rate.

In [25], for instance, Nassar et al. proposed two flexible electronics-based wearable sensors for plant growth and environmental monitoring. As in our study, the sensor with a dumbbell-like shape was used for measuring the elongation of the stem while the T and RH sensors (S_T_ of 0.0024·°C^−1^ and S_RH_ of 1.6·%RH^−1^) were integrated into a butterfly-shaped flexible patch attached to a leaf. Results of the strain sensor showed an elongation of 2.7 cm/day when placed on a barley plant leaf and an elongation of 905 μm/day when placed around the stem of a lucky bamboo.

Another study presented a multimodal plant healthcare sensor directly printed on the *Scindapsus aureus* leaves to measure growth and ambient T combined with light intensity and leaf hydration reaching interesting results: the leaf grew more in width than in length and more during the night than the day when T and light decrease, and consequently, the leaf hydration increases [29]. As in our study, both indoor and outdoor tests were carried out to assess the performance of the proposed system [29]. In our experimental conditions, the stem growth of tomato plants was measured only according to its longitudinal axis since the longitudinal elongation is primary compared to the diameter growth in tomato plants at the considered phenological stage for the tested time.

Differently from the state-of-the-art technologies, in this study, we proposed for the first time the use of FBG technology to provide an attractive solution for plant wearables’ development and applications. The FBG advantages of high sensitivity, multiplexing capability, and high resistance to harsh environmental conditions make the proposed technology particularly suitable for plant health monitoring or disease diagnosing through the development of plant wearable sensors.

The promising results of this study will foster future tests both inside the growth chamber and outdoors. Particular attention will be given to the use of FBG sensors (functionalized and bare gratings) on plant leaves, fruits and/or in the soil for furtherly investigating the influence of stressing conditions (e.g., salinity, thermal stress, and moisture stress) on the plant growth. This analysis will gain a deep understanding of plants’ responses to environmental stressors and soil properties and mitigate adverse conditions for their growth. Furthermore, other FBG-based flexible sensors will be developed to be easily multiplexed to the proposed plant wearables in an array configuration for better investigating the growth along different directions. For instance, another wearable sensor for *ε* measurement could be ringed around the stem to enable the monitoring of the radial growing simultaneously to the longitudinal elongation and further assess the presence of any anisotropic behavior of plants’ growth as suggested in [52,53].

## 6. Conclusions

This paper described an innovative system consisting of three plant wearable sensors based on FBG technology. This is the first attempt for the simultaneous monitoring of plant elongation and microclimate changes (i.e., T and RH in the surrounding environment) by using FBG sensors. The novel design of the proposed system together with the FBG advantages will play a crucial role in sowing the seeds of a new agricultural revolution: so-called Smart Farming. On a broader perspective, a similar system with its high multifunctionality and performance combined with image processing, big data, and cloud computing will allow for improving crop productivity and food security through advanced monitoring techniques suitable for applications in Smart Farming.

## Data Availability

The data presented in this study are available on request from the corresponding author.
